# Review of history and mechanisms of action of lactulose (4-O-β-D-Galactopyranosyl-β-D-fructofuranose): present and future applications in food

**DOI:** 10.1007/s13197-024-05997-z

**Published:** 2024-05-08

**Authors:** Vid Vičič, Ruža Pandel Mikuš, Blaž Ferjančič

**Affiliations:** 1 Chair of Biomedicine in Healthcare, Faculty of Health Sciences, University of Ljubljana, Zdravstvena pot 5, 1000 Ljubljana, Slovenia; 2 Jata Emona d.o.o, Agrokombinatska Cesta 84, 1000 Ljubljana, Slovenia; 3 Department of Food Science and Technology, Biotechnical Faculty, University of Ljubljana, Jamnikarjeva ul. 101, 1000 Ljubljana, Slovenia

**Keywords:** Lactulose, Functional food, Prebiotic, Gummy candy, Microbiota modulator, Hard candy

## Abstract

Lactulose is a synthetic disaccharide composed of galactose and fructose. Literature review of history, legal status and possible food applications of lactulose in functional foods, such as confectionery and beverages. In the colon, lactulose is fermented by the microbiota and acts as a selective modulator of bacterial growth, promoting the growth of *Lactobacilli* and *Bifidobacteria*. It generates organic acids, such as short-chain fatty acids and lactic acid, which lower the pH of the colon and act as an osmotic laxative. Lactulose was first used in 1957 as an ingredient in an infant formula. Later it was registered as a prescription drug and banned for food use in many countries. In 2012, lactulose received an EU (European union) health claim “contributes to acceleration of intestinal transit”. It can be used in food and food supplements across all age groups, from infants to the elderly. Lactulose has favourable technological properties, such as sweetness of 48–62% sucrose without an aftertaste, high solubility, low cariogenic potential and stability. Lactulose gummy candy, without added sweeteners, has an overall likability comparable to classic sucrose/glucose-based candy. With more than 60 years of safe use in infant, child, adult and elderly population, lactulose is an ideal ingredient for prebiotic functional food. Its technological properties allow for development of functional candy and beverages almost indistinguishable from those made from sucrose.

## Introduction

**Fig. 1:**
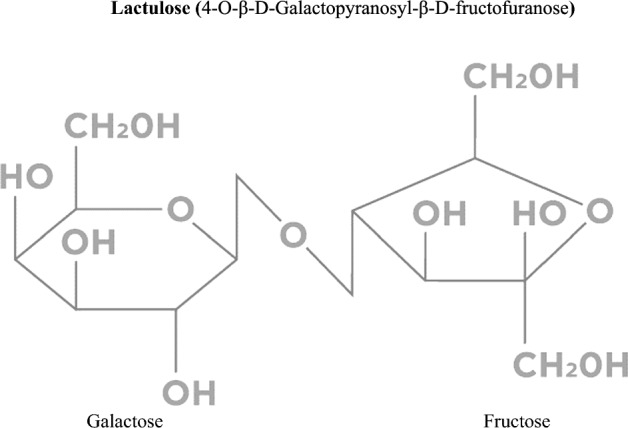
Chemical structure of lactulose

Lactulose (IUPAC: 4-O-β-D-Galactopyranosyl-β-D-fructofuranose) (Fig. 1) is a synthetic disaccharide consisting of galactose and fructose linked by a β-1,4-glycosidic bond (Panesar and Kumari 2011). Lactulose does not occur naturally, but it does occur in heat-treated milk. For this reason, it is used as a measure of the severity of heat treatment of milk products, especially UHT (Ultra-high temperature processing) milk (de Oliveira Neves et al. 2018).

Lactulose passes through the stomach and small intestine without being digested or absorbed in significant amounts. It reaches the colon intact, where it is fermented by the intestinal microbiota. It acts as a selective bacterial growth modulator by promoting the growth of bacteria that can use lactulose as an energy source. It promotes the growth of *Lactobacilli* and *Bifidobacteria* and inhibits the growth of *Bacteroides*, *Clostridia,* coliform bacteria, *Eubacteria* and *Salmonella*. The products of this fermentation are low molecular weight organic acids: lactic, butyric, acetic, formic and propionic acids, which lower the pH of the colon and inhibit the growth of microorganisms that cannot tolerate low pH, especially *Salmonella*. Low molecular weight organic acids are also osmotically active, increasing osmotic pressure in the intestine and acting as osmotic laxatives. Some organic acids are absorbed, consequently 1 g of lactulose provides 2 kcal of energy (9 kJ) (Schumann 2002).

Today, lactulose is mainly known as a drug for the treatment of constipation and hepatic encephalopathy (Schumann 2002), however, lactulose was first used in food. Its use in food began in 1957 when Petuely successfully used it in an infant formula, testing it on more than 300 infants and calling it the "bifidius factor". It was the first prebiotic ever discovered (Petuely 1957).

The aim of this article is to give a comprehensive review of the historical, present and future possibilities of using lactulose in food. We provide an extensive and practical review of the physiological mechanisms, technological properties and an overview of current developments in the market.

## Physiological effects of lactulose

**Fig. 2:**
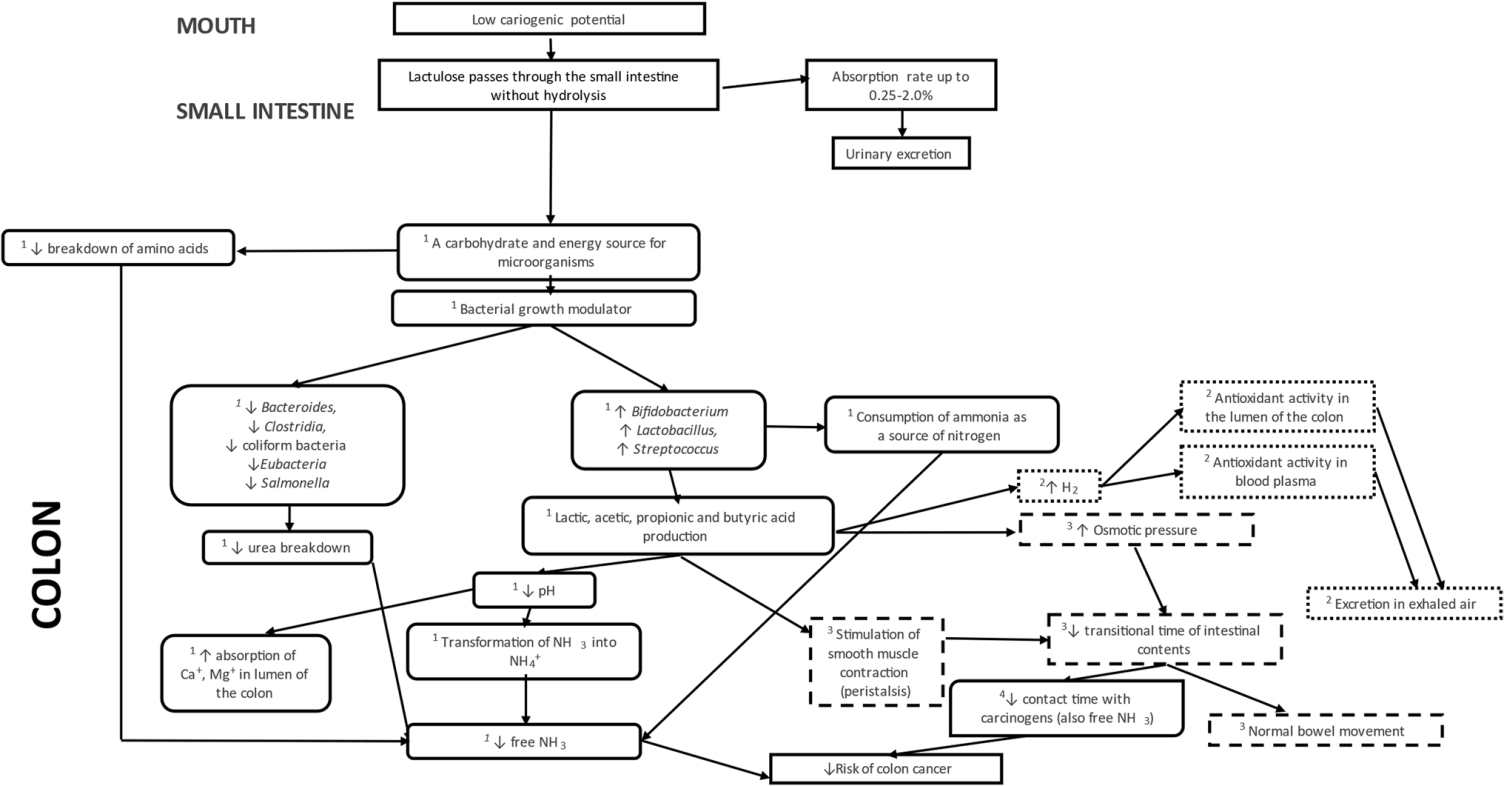
Overview of physiological functions of lactulose in human body. Legend: ^1^Lactulose as a substrate for microbial growth promotes the growth of *Bifidobacterium*, *Lactobacillus* and *Streptococcus* thus increasing the production of short-chain fatty acids and lactic acid. This results in decreased pH in the colon lumen, increasing absorption of Ca^+^ and Mg^+^. At the same time, the consumption of lactulose decreases free ammonia in the colon lumen by reducing the rate of breakdown of free amino acids for carbon needs of bacterial growth. Already present free ammonia and urea are used as nitrogen sources for bacteria, resulting in lower net values. Last but not least, due to lower pH, transformation rate from ammonia to ammonia cation is increased (Schumann 2002). ^2^Consumption of lactulose increases H_2_ production in the colon lumen. It was shown that molecular hydrogen is an exceptionally potent antioxidant. Due to its size, it can pass through cell membranes and react with ROS (reactive oxygen species) inside the cell and nuclei. Surplus of hydrogen is excreted in exhaled air (Chen et al. 2011). ^3^Lactulose enters the colon unchanged, causing an increase in osmotic pressure (osmotic pressure is also increased by SCFAs) leading to increased amount of water in the colon lumen. This mechanism complements the effect of SCFAs that stimulate smooth muscle contractions. The synergy of mentioned mechanisms decreases transitional time of the colon bolus, enabling normal bowel movement in constipated patients. ^4^A faster transitional time leads to a reduced exposure to carcinogens in the colon, thereby reducing the risk of developing colon cancer (Dahl and Stewart 2015)

The physiological effects of lactulose are most pronounced in the human colon, since lactulose cannot be hydrolysed by human enzymes (Schumann 2002). One of the simplest physiological effects of lactulose is the prevention of constipation. The presence of lactulose in the colon increases osmotic pressure, allowing water to enter the colon lumen and facilitating defecation (Aït Aissa and Aïder 2013). Kot and Pettit-Young (1992) reported lactulose as laxative, that does not cause dependency. However, as shown in the Fig. 2, lactulose has more than just this physiological function. A small daily intake can decisively promote the growth of probiotic bacteria, mainly *Bifidobacteria* and *Lactobacilli* (Bouhnik et al. 2004). This selective metabolism of lactulose does not only promote the growth of beneficial bacteria, but it also enables these bacteria to produce SCFAs (short-chain fatty acids) by fermenting lactulose. The fermentation products are acetate, propionate, and butyrate. Lactic acid is also produced, but is converted to butyrate by the colon microbiota. Members of *Bifidobacteria* and *Lactobacilli* produce acetate and lactic acid, which serve as a substrate for other bacteria and are converted to butyrate. SCFAs are rapidly absorbed by the colon epithelium and used as a substrate for respiration. Butyrate is the preferred energy source for colonocytes (Karakan et al. 2021). More recent research found the possible link between lactulose promoting growth of probiotic bacteria in colon with its laxative effect. Improving colon microbiota results in increased SCFA production, which promotes bowel movement by releasing serotonin. This mechanism of constipation prevention acts in synergy with increased osmotic pressure in colon (Ma et al. 2023).

In addition, SCFAs play multiple roles in human health. The presence of SCFAs and lactic acid in the colon lumen lowers pH, which enhances the absorption of minerals, mainly Ca^+^ and Mg^+^ (Brouns et al. 2002). Decreased pH in the colon lumen allows the conversion of free ammonia to NH_4_ (Topping and Lockett 2016). This mechanism, along with the use of ammonia as a nitrogen substrate for growth by colonic bacteria and selectivity in promoting probiotic bacteria, is the main reason for the use of lactulose as a treatment for long-term hepatic encephalopathy (Hudson and Schuchmann 2019).

The action of SCFAs is not limited to the colon lumen, as they are absorbed into the lamina propria and further into the bloodstream. This makes SCFAs signalling molecules (Kumar et al. 2020). In this way, SCFAs influence the production of satiety hormones (PYY (peptide tyrosine tyrosine) and GLP-1 (glucagon-like peptide-1)) (Tannock and Liu 2020). SCFAs also play a role in modulating the human immune system. Inflammatory processes are downregulated, while immunoglobulin A is upregulated. T-helper cell response is also increased (Tannock and Liu 2020). In addition, butyrate inhibits tumour cell development by inhibiting histone deacetylase while upregulating glutathione S-transferase, one of the most potent antioxidants (Scharlau et al. 2009). Considering the antioxidant activity, it is important not to overlook the mechanism of hydrogen generated during lactulose fermentation. Free hydrogen can cross cell membranes and act as an antioxidant, neutralizing oxygen radicals in human cells (Chen et al. 2011).

## History and legal status

Lactulose was first synthesised from lactose by Montgomery and Hudson in 1930 (Montgomery and Hudson 1930). In 1943, Bessau found that the growth of *Lactobacilli* in the large intestine is promoted by "caramelised lactose" (Haemmerli and Bircher 1969). In 1957, Petuely synthesised and purified lactulose and successfully used it in an infant formula. Tests were carried out on more than 300 infants. He found that 1.2 g of lactulose per 70 kcal (294 kJ) of infant formula produced a pH environment and a microbiota of *Lactobacilli* and *Bifidobacteria* similar to that of breastfed infants. He referred to this as the "Bifidius factor". Thus, lactulose was the first prebiotic ever described (Petuely 1957). Considering that the mother's milk intake in the first month of an infant's life is ~ 624 mL/day (Rios-Leyvraz and Yao 2023), this would correspond to ~ 6.25 g lactulose/day. Only three years later, it was marketed by the Japanese company Morinaga milk as an ingredient in infant formula. It is still used today (Morinaga milk 2023). The use in infant formula with relatively high dosage indicates that lactulose is very safe (Petuely 1957). The LD50 (median lethal dose for rat) for lactulose is 18.16 g/kg of body weight (British pharmacopoeia 2013).

As a treatment for constipation, lactulose was first described in 1959 (Mayerhofer and Petuely 1959), the first double-blind clinical trial confirming its efficacy was conducted in 1968. The authors pointed out that constipation often requires prolonged treatment. Therefore, the “intestinal regulator”, as referred to by the authors, should be carefully selected to provide a gentle effect without systemic effects (non-absorbable), without adverse effects such as cramps or electrolyte depletion, and without toxic or habit-forming properties. Lactulose certainly meets these criteria. The only observed side effects were temporary intestinal distension and gas formation (Wesselius-De Casparis et al. 1968).

In 1964, Hoffmann and others discovered that *Bifidobacterium, Lactobacillus* and *Streptococcus* can break down lactulose into lactic acid and other organic acids through strong fermentation. *E. coli* and *Staphylococcus aureus* could only weakly metabolise lactulose. *Ristella*, *Proteus*, *Salmonella* and *Shigella* were not able to metabolise lactulose (Hoffmann et al. 1964). In 1975, Hoffmann discovered that the breakdown of lactulose in the colon lowers the pH, creating an unfavourable environment for *Salmonella* and provides an effective alternative to antibiotics for eradicating *Salmonella* in healthy carriers (Hoffmann 1975).

As a treatment for hepatic encephalopathy, lactulose was first described by Bircher et al. (1966). There are several mechanisms for this, including lowering pH, promoting the growth of bacteria that consume ammonia, and facilitating faster passage of intestinal contents (Bircher et al. 1966; Haemmerli and Bircher 1969).

In 1977, lactulose was approved in the USA as a prescription-only drug for the treatment of constipation and hepatic encephalopathy (Mukherjee and John 2023). Its use in food has been banned. Schumann states in 2002 that lactulose is classified as a medicinal product and not as a food ingredient, despite its very close physiological and chemical relationship with other prebiotics. At the time of his review (2002), it was only used in food in Japan, the Netherlands and Italy (Schumann 2002).

In Europe, this changed in 2012 when lactulose was given the EU health claim "lactulose reduces intestinal transit time", based on an intake of 10 g of lactulose (Table 1). Currently, there are no restrictions on the use of lactulose in food in the EU. It can be used for its laxative or technological effects in foods, including food supplements, across all age groups, from infants to the elderly (EFSA 2010; European Commission 2012).

**Table 1:** EFSA (European food safety authority)’s health claim on lactulose (European Commission 2012)

Nutrient, substance, food or food category	Claim	Conditions of use of the claim	Conditions and/or restrictions of use of the food and/or additional statement or warning	EFSA Journal number
Lactulose	Lactulose contributes to an acceleration of intestinal transit	The claim may be used only for food which contains 10 g of lactulose in a single quantified portion. In order to bear the claim, information shall be given to the consumer that the beneficial effect is obtained with a single serving of 10 g of lactulose per day	**–**	2010;8(10):180

## Technological properties and considerations

Lactulose as an ingredient is mainly sold as a liquid (syrup) or in the form of crystals. Lactulose as a liquid is a sweet, clear, yellowish, odourless syrup (Shendurse and Khedkar 2016). According to the European Pharmacopoeia, it must contain at least 62 g/100 mL lactulose and may contain smaller amounts of other sugars, including lactose (< 10%), epilactose (< 10%), galactose (< 15%), tagatose (< 4%) and fructose (< 1%) (Council of Europe 2007).

Lactulose crystals are a white, odourless crystalline powder with a sweet taste and have a sweetness level ranging from 48 to 62% of sucrose and are approximately 150% sweeter than lactose (Parrish et al. 1979). It is soluble in water, insoluble in ether and slightly soluble in methanol. At 30 °C, the solubility of lactulose in water is 76.4% (w/w) and increases to 86% at 90 °C. The melting point is 168.5–170.0 °C (Shendurse and Khedkar 2016). It readily caramelises (Kerling 1977; Weterings and Pluim 1993; Lisitsyn et al. 2016) and can even be made into toffee (Kerling 1977).

Glass transition temperature (Tg) of lactulose is 94.7 °C (Ngono et al. 2019). For comparison, the Tg of sucrose is ~ 60 °C (Roe and Labuza 2005). This has practical implications, e.g. in the production of hard candy, where it means that the process has to be carried out at higher temperatures to maintain the liquid state (Vičič 2022). Amorphous lactulose, obtained by spray drying (Lisitsyn et al. 2016) or (vacuum) evaporation followed by cooling and grinding (Weterings and Pluim 1993), is very hygroscopic (Weterings and Pluim 1993; Lisitsyn et al. 2016). Despite the high cost of lactulose crystals, this could be the reason why no products of such nature are available.

Lactulose can be metabolised by microbiota in oral cavity, but has a much lower acidogenic potential compared to sucrose (Mäkinen and Rekola 1975; Moynihan et al. 1998), therefore it could be used instead of sugar in chewing gum.

## Latest innovations

**Fig. 3:**
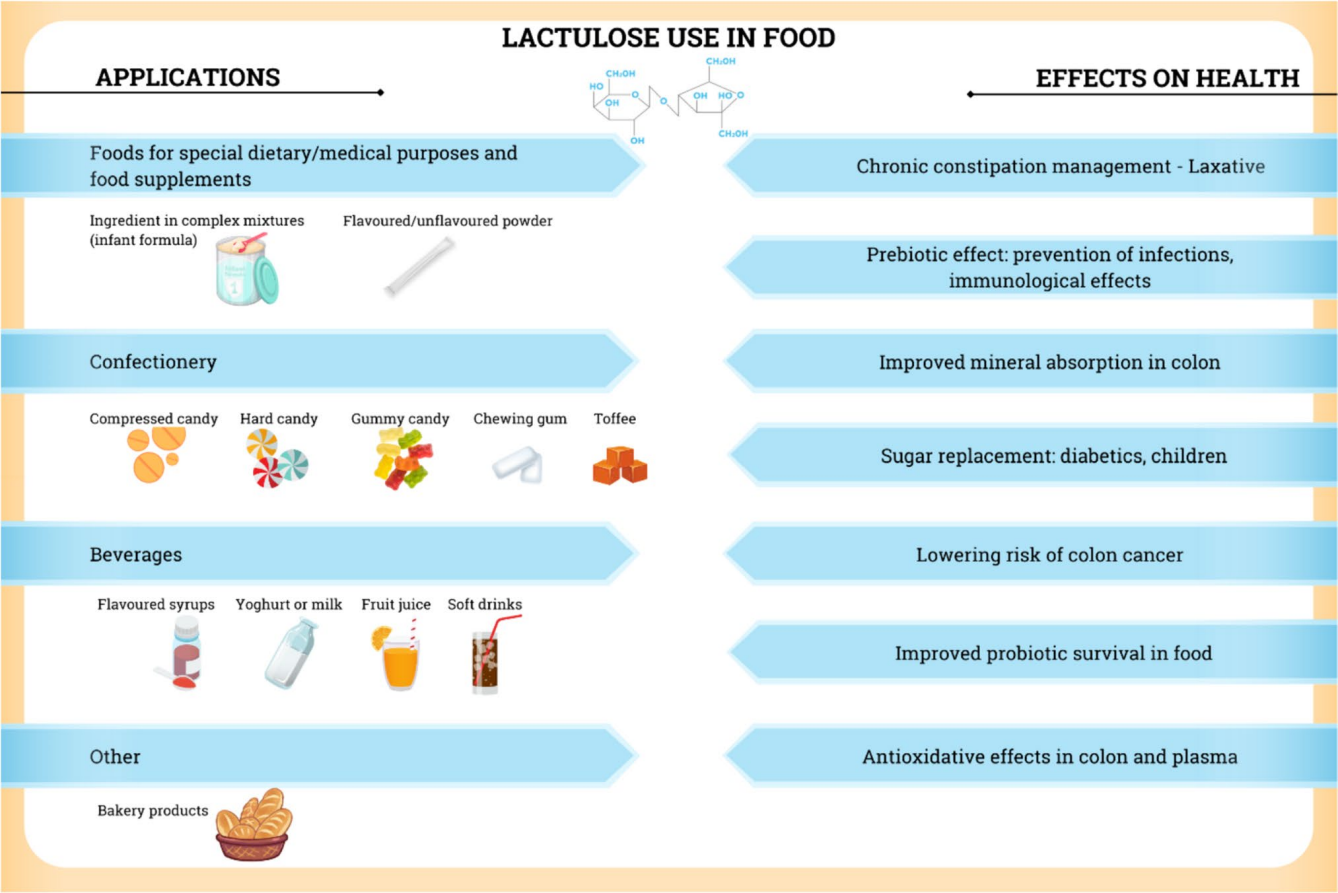
An overview of the use of lactulose in food

Most lactulose is consumed as a medicine in the form of unflavoured or flavoured lactulose, which is available in both liquid and crystal forms. In food, lactulose is primarily used as an ingredient in infant formula, yoghurt, and soft drinks (Seki and Saito 2012). For an overview of possible food applications, see Fig. 3.

### Yoghurt and milk beverages

Yoghurt containing lactulose as a prebiotic was first described in 1986 (Olano et al. 1986; Porkka et al. 1988). In addition to prebiotic/laxative effect for the consumer, lactulose increases survivability of probiotic strains (Jooyandeh et al. 2023). Yoghurt and kefir containing lactulose are currently present on Ukrainian market under brand Lactonia (Lactalis 2024). In 2023, Danone South Korea launched Activia Gold, a "symbiotic" product consisting of a milk-based product with lactulose and probiotic pills enclosed in the cap (Solactis 2022).

### Gummy candy

In 2017, Abbott Laboratories (USA) introduced the gelatine-based gummy cand "Dupha Bears" and "Dupha Chews" as dietary supplements (Roland 2017). In 2020, Abbott introduced pectin-based "Duphalac bears" in India, the product is labelled as "prebiotic food. Not for medical use"(HealthWord 2020). In 2019, Jata Emona (Slovenia, EU) introduced a similar gelatine-based gummy candy under the "Laxemon" brand in the food supplement category (Česen 2020; Jata Emona 2023)*.*

**Fig. 4:**
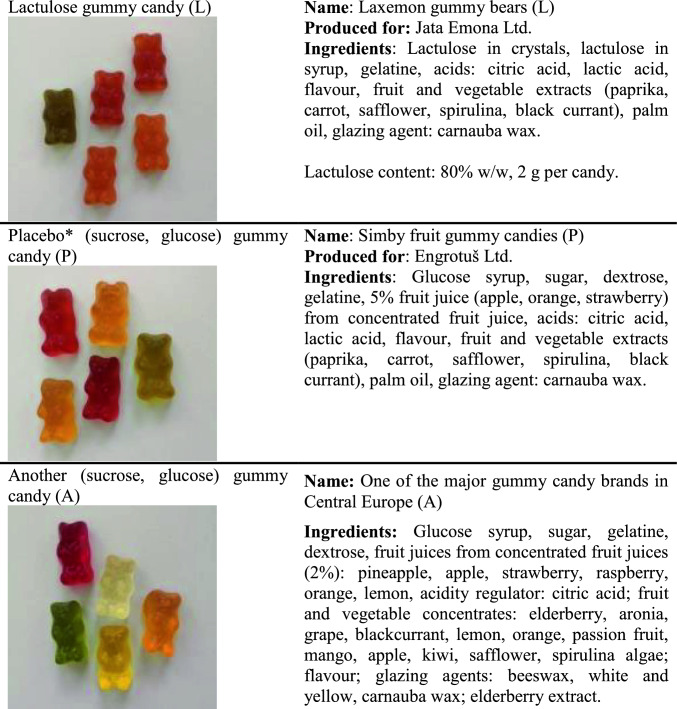
Visual comparison of lactulose-based gummy candy and sucrose/glucose placebo. Published with the author’s permission. The gummies were manufactured in the same production facility using identical moulds and ingredients, with the exception of fruit juice concentrates, which were excluded in the lactulose candy. Published with the author’s permission (Česen 2020)

The literature on this topic is limited, with only a comparison of lactulose gummy candy with sucrose and glucose candy being conducted thus far by Česen in Slovenia (2020). With the author’s permission, we are publishing the translated results. Česen (2020) compared the technological properties, sensory properties, chemical composition, and laxative effect of lactulose gummy candy "Laxemon" with placebo sucrose/glucose-based gummy sweets produced on the same production line with identical additives (gelatine, colour, flavour). Figure 4 illustrates that there are minimal visual differences between the samples.

**Fig. 5:**
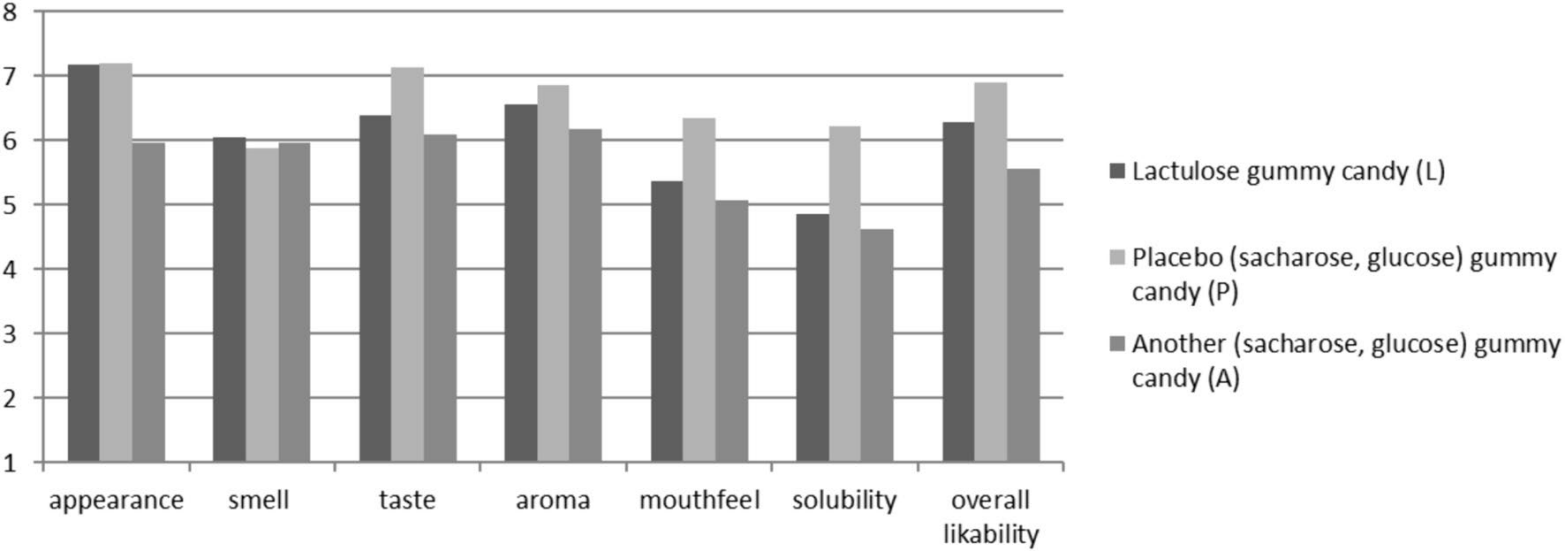
The sensory test was performed on 38 students aged 22–25, studying Food Technology (MSc) and Nutrition (MSc). Out of the participants, 34 were women. The scale used for rating the sensory attributes ranged from 1 (worst) to 9 (best). Published with the author’s permission (Česen 2020)

In the paired comparison test, it was found that 77% of participants preferred the placebo gummy candy (sucrose, glucose) and 23% of participants preferred the lactulose gummy candy. However, the comparison to a popular gummy candy brand (A) shows that the differences are still acceptable to consumers (Česen 2020). Detailed results are presented in Fig. 5.

**Fig. 6:**
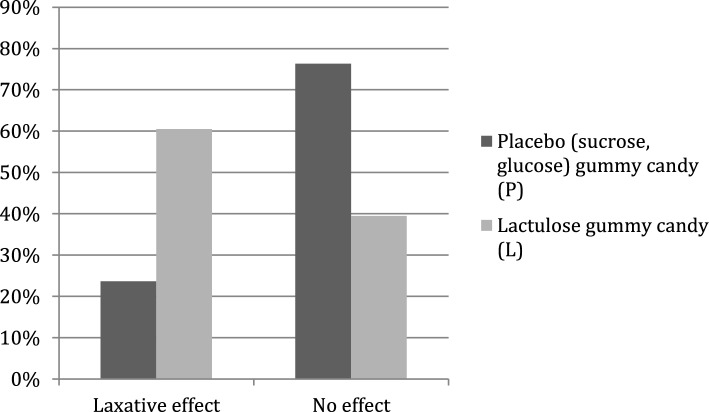
Laxative effect of lactulose gummy candy (L) containing 10 g of lactulose compared with placebo gummy candy (sucrose, glucose) (P). Published with the author’s permission (Česen 2020)

After consuming a gummy candy containing 10 g of lactulose, 60.53% of the subjects reported perceiving a laxative effect. In contrast, after consuming the placebo, only 23.7% of the subjects reported perceiving an effect (Fig. 6).

### Hard candy

In 2022 a patent for lactulose-based hard candy was granted (Vičič 2022). In this patent, the process involves heating the lactulose syrup to evaporate the moisture and then adding flavourings and colourings. The resulting mixture is then poured into moulds, cooled, and coated with oil, wax, or starch before being packaged. The main difference in technology compared to sucrose/glucose-based sweets is the higher process temperature, as the mass needs to be around 130–140 °C when poured into moulds. This is necessary due to the higher glass transition temperature of lactulose (see previous chapter). Hard candies have a significantly longer shelf life and better temperature stability compared to gummy candies, as gelatine-based gummy candies melt irreversibly at temperatures around 35 °C (Tireki et al. 2021). Compared to compressed lactulose candy made from lactulose crystals, using liquid lactulose can offer cost-efficiency advantages (Vičič 2022).

## Stability in food

Stability during heat treatment at low pH is crucial for the successful use of any substance in beverages and confectionery. Shendurse and Khedkar found that lactulose is "stable and hardly decomposes when heated for 10 min at 130 °C and a low pH value” (Shendurse and Khedkar 2016). Seki and Saito (2012) also mention high thermostability in acidic conditions. However, no reference or experimental data was provided to support this claim. Lardieri with others (2016) evaluated the stability of lactulose added to milk, juice and soda after 72 h. The authors concluded that the stability of lactulose in milk, juices and soda could not be determined due to the interference of naturally occurring sugars, which affected the detection of lactulose (Lardieri et al. 2016). Since lactulose is produced by heating lactose in the presence of alkali (Panesar and Kumari 2011), it can be inferred that lactulose is heat stable. However, no published data on the stability of lactulose during storage were found.

## Conclusion

Lactulose, which was the first prebiotic ever discovered, has been utilised as a prebiotic in infant formula. Today, lactulose is recognised as a medicine used for the treatment of constipation and hepatic encephalopathy. Due to this fact, as well as legislative limits in some parts of the world, the use of lactulose in food has often been restricted.

Even a small daily intake of lactulose has an impact on the growth of probiotic bacteria, particularly *Bifidobacteria* and *Lactobacilli*. This leads to an increased production of short-chain fatty acids and lactic acid. As a result, there is improved absorption of Ca and Mg. Through various mechanisms, the consumption of lactulose exerts antioxidant and anticarcinogenic effects. In Europe, lactulose received a health claim in 2012 for accelerating intestinal transit. It can be used without restrictions or limitations in all population groups. Due to its favourable technological properties, lactulose can be utilised as a sugar replacement in a wide range of food products, including gummy candy, hard candy, fruit juice, soft drinks and milk products.

## Data Availability

Not applicable.

## Abbreviations

EU: European union
UHT: Ultra-high temperature processing
SCFAs: Short-chain fatty acids
ROS: Reactive oxygen species
PYY: Peptide tyrosine tyrosine
GLP-1: Glucagon-like peptide-1
LD50: Median lethal dose
EFSA: European food safety authority
Tg: Glass transition temperature
